# COVID-19, Fake News, and Vaccines: Should Regulation Be Implemented?

**DOI:** 10.3390/ijerph18020744

**Published:** 2021-01-16

**Authors:** Julio Emilio Marco-Franco, Pedro Pita-Barros, David Vivas-Orts, Silvia González-de-Julián, David Vivas-Consuelo

**Affiliations:** 1 Research Centre for Economics Engineering, Polytechnic University of Valencia, 46022 Valencia, Spain; jemarfra@doctor.upv.es (J.E.M.-F.); dvivas@upv.es (D.V.-C.); 2 Doctoral Programme of Rule of Law and Global Governance, Faculty of Law, University of Salamanca, 37007 Salamanca, Spain; 3 Nova School of Business and Economics, Nova University, 2775-405 Carcavelos, Portugal; ppbarros@novasbe.pt; 4 Faculty of Law, University Pompeu Fabra, 08024 Barcelona, Spain; david.vivas@upf.edu

**Keywords:** COVID-19, vaccination, fake news, medical code of ethics, governmental regulations

## Abstract

We analysed issues concerning the establishment of compulsory vaccination against COVID-19, as well as the role of misinformation as a disincentive—especially when published by health professionals—and citizen acceptance of measures in this regard. Data from different surveys revealed a high degree of hesitation rather than outright opposition to vaccines. The most frequent complaint related to the COVID-19 vaccination was the fear of side effects. Within the Spanish and European legislative framework, both compulsory vaccination and government regulation of FN (Fake News) appear to be feasible options, counting on sufficient legal support, which could be reinforced by additional amendment. However, following current trends of good governance, policymakers must have public legitimation. Rather than compulsory COVID-19 vaccination, an approach based on education and truthful information, persuading the population of the benefits of a vaccine on a voluntary basis, is recommended. Disagreements between health professionals are positive, but they should be resolved following good practice and the procedures of the code of ethics. Furthermore, citizens do not support the involvement of government authorities in the direct control of news. Collaboration with the media and other organizations should be used instead.

## 1. Introduction

With the appearance of vaccines against COVID-19, it is worth asking whether their administration should be maintained as voluntary, which in turn raises the question of to what extent individual freedom can and should prevail over the common social good. On the one hand, this is a question that is rooted in community values (for example, in the USA, the value of the sovereignty and total autonomy of the individual is strongly rooted) and on the other, the concept of public health and the measures and institutions that are needed to take care of it, necessitating an analysis of how to make the corresponding regulations compatible with the individual values described.

The situation that arose after the outbreak of COVID-19, with lockdown measures and extra time for the generation, circulation, and reading of all kind of news, has been the perfect breeding ground for the development of denialist positions, conspiracy theories, and fake news (FN), which have sown turmoil in a part of the population that is facing a change of the social paradigm set after the Second World War and that seemed immutable.

The issue is particularly critical when misinformation or opinions that go against the flow of evidence-based information are publicized by expert professionals, as opinions of scientists, doctors, other health experts are generally considered very trustworthy [1,2,3].

However, political action must be based on good global governance, and good governance requires public legitimacy [4,5,6], something that is complicated when negative attitudes without a scientific basis become widespread and are even promoted by some health professionals.

This paper overviews the issue of compulsory vaccination when faced with misinformation concerning COVID-19, FN, and myths related to vaccination, with particular emphasis on those publicized by healthcare professionals or experts, analysing the legal bases for eventual regulatory control and its acceptance by citizens.

## 2. Materials and Methods

Following the legal reasoning (comprehensive *Juristischen Methodenlehre*) [7,8] we analyse the autonomy of the individual and the freedom of information as essential elements of the freedom of expression, the fundamental, human, and conventional rights versus the compulsory introduction of the COVID-19 vaccine, and the possible regulation of published news. Public opinion input, as promoted by the Organisation for Economic Co-operation and Development (OECD/OCDE) and the EU [9], has been obtained as part of the current request for evidence in the good governance approach for policymakers [4,5,6,10,11].

Citizens’ opinions, from different surveys and countries related to FN in general and to COVID-19 in particular, have been obtained and processed (grouping and proportional computing [12]), after being collected through Statista^®^ (Statista GmbH, Hamburg, Germany) [13]. Specific information for the EU, including the Flash Eurobarometer 464, has also been used to gather information [14]. Earlier references include the report for the European Commission (State of vaccine confidence in the EU) [15] and the IPSOS survey for the World Economic Forum on vaccination to prevent COVID-19 [16]. The latest data on voluntary vaccination intentions in Spain come from the Centre for Sociological Research of Spain (CIS) [17], and from the Invymark Institute, as presented in the two newscasts on the television channel *La Sexta* on 28 November 2020.

Recent documents and regulations from the EU and Spain have also been analysed, including information provided at the webinar (University of Salamanca, 26 November 2020) *El Procedimiento de actuación contra la desinformación en la defensa del Estado de Derecho* (Procedure for action against disinformation in defence of the rule of law) by the Director of the Department of National Security of the Presidency of the Government.

## 3. Results

**Figure 1:**
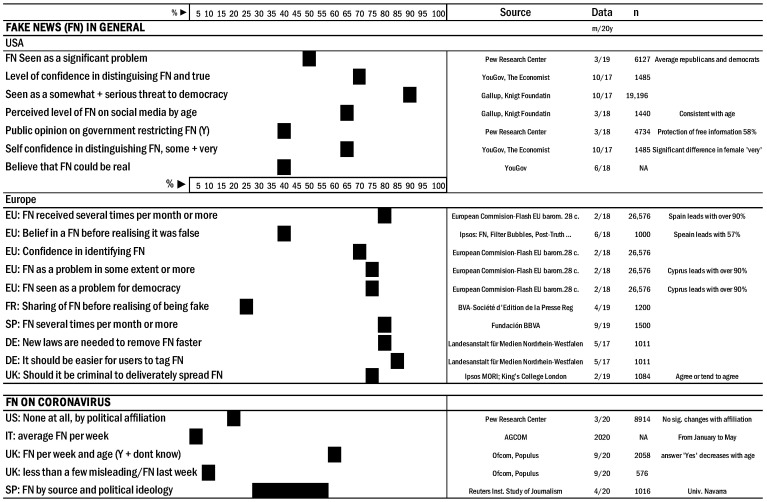
Summary of citizen opinion from different countries related to false news, including fakes on COVID-19. Source: Authors’ computation from different surveys procured from the Statista da-tabase.

The legal analysis will be commented on in the discussion section. Public opinions related to FN are summarized in Figure 1. Surveys suggest that citizens do not much trust in information from the media but feel reasonably confident in their capacity to distinguish fake news from real.

The most frequent complaint, related to vaccination against COVID-19, is fear of side effects [15,18], which raises the question of how safe and effective the vaccine should be, and the procedures to counteract this fear, mostly developed after the confusion of ideas resulting from the constant circulation of FN.

FN is considered a major problem in 68% (65 or older) to 80% (18 to 29 years old) of USA citizens, and somewhat of a threat or a serious threat to democracy in 88% (Knight Foundation Gallup) [19]. The perceived level of FN on social media in the USA, as of May 2018, is quite consistent over all age groups (about 63%). Social media sites are considered partly or mostly responsible for the spread of FN in 89% of the cases, with 69% considering that these social media sites are not doing enough to stop the spread of FN on their sites (Monmouth University) [20,21].

As for Europe and according to data from the Flash Eurobarometer as of February 2018 (*n* = 26,576), about 80% of the respondents encountered FN several times a month or more, with 37% of responses in the everyday/almost everyday group [14].

About 81% of Spaniards (2019) perceived FN “often” or “several times”, with only 7% indicating almost never [22], meaning the great majority of the population is perceiving a significant amount of FN.

In Italy, the share of online FN related to coronavirus between January and May 2020 was about 5% per week, with Facebook recognized by almost 80% of those surveyed, regardless of age, as the primary social media in this regard [23,24].

In the UK (September 2020), 20–30% of respondents had encountered information/news about coronavirus that they thought was false or misleading in the previous week, with a similar number saying they did not know, leaving less than 50% declaring they were unaware of false information [25]. The pattern is consistent when analysed by age groups; 81% declared that received FN a few times a week or more, with 7% of them reporting ten or more times a day [25].

The issue of misinformation has become so critical that the World Health Organization (WHO) has dedicated a site specifically to reporting misinformation online [26]. The European Commission also recommends following the advice of public health authorities, and websites of relevant organizations. The European Centre for Disease Prevention and Control (ECDC) and the WHO work in close cooperation with online platforms [27,28,29]. There are also several funded projects for fighting against disinformation [30] and a permanent analysis by the Social Observatory for Disinformation and Social Media Analysis (SOMA) [31].

**Figure 2:**
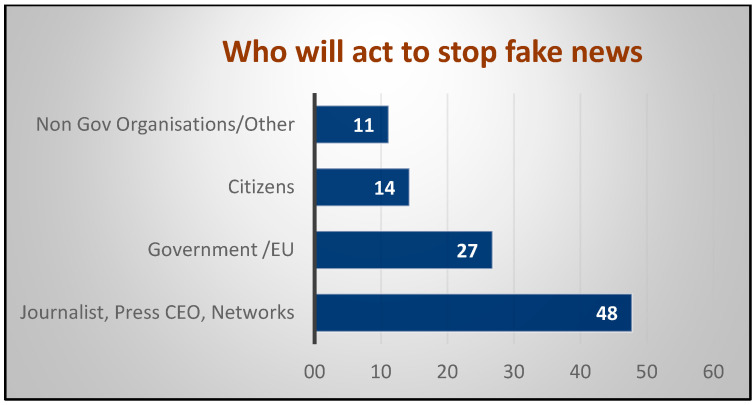
Who will stop fake news? (EU 2018). Source: Authors’ mathematical computation of data from Flash Eurobarometer 464 [14].

A 2018 EU survey on FN and disinformation online [14]—where up to three answers could be chosen from seven possible options—requested the opinion of respondents on who should act to stop the spread of FN. After mathematical processing of the resulting 224 points as relative percentages, they were included in four main groups: (1) mass media professionals plus administrators or media organizations, (2) governmental authorities either at a national or EU level, (3) citizens, and (4) non-governmental organizations + other, with the predominant option to stop FN being for the professionals to act themselves (Figure 2).

In a 2018 a survey about vaccines with 28,782 respondents across the 28 EU member states, the perception towards vaccines was positive, with agreement (strongly or tend to agree) at 90%, safety at 82.8%, effectiveness at 87.8%, and compatible with religious beliefs at 78.5% [15]. The importance of vaccination was found to be related to the disease.

According to the IPSOS survey for the World Economic Forum (24 July to 7 August 2020), 74% of respondents agree (37%) or somewhat agree (37%) on the question as to whether they will get the vaccine when available. Forty percent thought they would not have the ability to get the vaccine by the end of 2020. Among the reasons for not getting vaccinated, 56% were worried about side effects, 29% think that it will not be effective, 19% that they are not enough at risk from COVID-19, and 17% are against vaccines in general [16].

The perceptions and intentions of the Spanish population regarding the COVID-19 vaccine have been analysed by the Centre of Sociological Investigation of Spain (CIS) on a regular basis. The number of respondents willing to be vaccinated fell from 44.4% in September to 32.5% in November, when 55.2% of overall respondents chose the option of waiting to see the side effects, but among those within the 18–24 age range, 72.3% of the cases preferred to wait [17]. Another survey from the Invymark Institute, during the week of 23 November, was broadcast by the television channel *La Sexta* (28 November 2020). Up to 61.6% among the groups surveyed do not believe that there will be effective vaccines in the coming months. Almost half of the respondents are not willing to be vaccinated when the vaccine is available (a similar percentage as in the CIS survey), with the reasons being, firstly, that they prefer to wait a while (50.1%), secondly, concern about side effects (44.4%), and, thirdly, that they do not believe vaccines are efficient (4.6%). Although there are some variations of the percentages in relation to the IPSOS survey data related to Spain, a substantial amount of the respondents expressed a high degree of hesitance rather than outright opposition to vaccines.

The lack of confidence in prevention is also evident in the response to the question (Invymark Institute) on when the respondent believes that a certain normality will return. The majority response (48.6%) is two or more years, for 42.0% in one year, and 8.8% believe that normality will not return for many years.

## 4. Discussion

As has happened in other critical phases of history, a percentage of a disoriented population is vulnerable to the influence of gurus and prophets who reveal the errors of today’s society and show alternative paths, alien to the official postulates. The spread of the SARS-CoV-2 virus is the perfect storm for the growth of conspiracies, flat-earthers, and such like.

The situation has significantly worsened since the COVID-19 pandemic included lockdowns, with resultingly more time spent on chatting and tweeting. A survey by the Reuters Institute for the Study of Journalism revealed that the percentage of the population that has found FN or misleading news related to coronavirus in Spain, as of April 2020, is quite consistent, even considering political ideology, although the data show a relevant increase in the percentage of right/far-right respondents’ attribution to the National government or linked organizations. The Institute also recognizes that “journalists no longer control access to information, while greater reliance on social media and other platforms gives people access to a wider range of sources and “alternative facts”, some of which are at odds with official advice, misleading, or simply false” [1].

At the present time, without effective antiviral treatment, social isolation and vaccination seem to be the most rational hope for fighting the pandemic. With this “RMS Titanic orchestra” social attitude briefly outlined; two key questions arise in this situation:

(1) Can the authorities implement these measures, for example, the vaccination of people who refuse it, even by use of force?
(2) Who (and how) should stop the publication of FN, particularly when this false news is published by registered health professionals, denying vaccination, and placing not only themselves at risk but the entire community? Is it legally and democratically founded to take coercive action against the desire of citizens and to act to curb the opinions of certain professionals who move outside the parameters of professional praxis?

Starting with this second point, in Spain, as in other developed countries, freedom of expression and the dissemination of news is a constitutional right (Art. 20 of Spanish Constitution [32]). Of even more importance, the constitutional text does not require the information to be true. This freedom is also supported by the European Convention on Human Rights (ECHR) (Art. 10) [33], although both texts establish limitations in relation to public safety (ECHR, Art. 10.2), the observance of the other rights recognized in Title I of the Spanish Constitution (Art. 39.1), and the protection of public health (Art. 43).

The problem of misinformation has grown to a point that the EU has promoted an in-depth analysis. “The legal framework to address ‘fake news’: possible policy actions at the EU level” [34] and reinforced responses in collaboration with the USA [35]. Furthermore, the EU is strengthening actions to tackle COVID-19 disinformation [36], as “in the EU and elsewhere, coordinated disinformation messaging seeks to frame vulnerable minorities as the cause of the pandemic and to fuel distrust in the ability of democratic institutions to deliver effective responses” (EEAS special report on 1 April 2020, recently updated) [37]. The document also includes a taxonomy of the misinformation relating to the COVID-19 pandemic.

Although the initial efforts of both European and Member States’ authorities were devoted to combating specific misinformation in order to ensure the transparency of electoral processes, the growth of FN and the subsequent erosion of institutions and the danger of polarization of the society that this entails have forced states to take measures, which in the case of Spain, have been specified in the Official Gazette of 5 November 2020, with Order PCM/1030/2020, of 30 October, which publishes the Procedure for Action Against Disinformation approved by the National Security Council [38].

This new regulation will allow the collaboration of the media in the fight against disinformation that may cause damage or affect fundamental rights, such as the right to health as enshrined in law. As for COVID-19 and cybercrime, according to the EU report: “Criminals use the pandemic to carry out various scams and attacks […]. Europol, the EU’s law enforcement agency, collects information from EU member states and publishes regular reports on how criminals are adapting their crimes to exploit the coronavirus pandemic” [39].

As result of misinformation, a citizen may act (or omit to act) with dangerous consequences for him/herself or for third parties. This is even more the case when it refers to behaviour outside of the medical praxis related to the COVID-19 vaccine, under the influence of a publication of an (allegedly) qualified healthcare professional, given that, in Spain, scientists, doctors, and other health experts rank high in the degree of confidence in the information they publish [1].

The question of misinformation provided by medical professionals has two key aspects:

(1) There is freedom in receiving and publishing information about health, but this must also be reliable as required by standards of praxis and *lex artis ad hoc*. Freedom of information is an essential element of the fundamental right of freedom of expression, as recognized by Resolution 59 of the UN General Assembly adopted in 1946 [40], as well as by Article 19 of the Universal Declaration of Human Rights (1948) [41]. According to the World Medical Association Declaration of Geneva (1948), medical knowledge cannot be used to violate human rights and civil liberties, even under threat [42].

The right to have proper information relating to health is also recognized in the Protection of Human Rights and Dignity of the Human Being with regard to the Application of Biology and Medicine (Oviedo, 4 April 1997) [43].

The information provided by Spanish physicians must be understandable and true (Art. 4.2) of Law 41/2002, establishing the basic regulation of patient autonomy and of rights and obligations regarding clinical information and documentation. Additionally, in Art. 6., they “have the right to know about the health problems of the community when these involve a risk to public […]” [44]. This is a derivative of the human rights recognized in international treaties and the Spanish Constitution (health protection (Art. 43) and education of consumers and users (Art. 51)) [32].

(2) The practice of medicine is a regulated activity. It is subject to compulsory membership of an Official Medical Association (*Colegio Médico Oficial*) and compliance with its rules and procedures, mainly the ethical code [45]. The medical association can initiate disciplinary proceedings (Art. 44.4) and impose the sanctions regulated in the General Statutes of the Medical Association (Art. 2).

The code of ethics establishes in Art. 7 that “a medical act is understood to be any lawful activity carried out by a legitimately qualified medical professional, whether in the field of care, teaching, research, expertise, or others […]” Therefore, the release of information is included in this concept. A number of other articles in this code focus on avoiding the imposition of their own convictions (Art. 9.1), assuming the negative consequences of their actions and errors, offering a clear, honest, constructive, and adequate explanation (Art. 17.1), providing assistance of human and scientific quality (Art. 21.1), refraining from actions that exceed their capacity (Art. 22), and avoiding “practices inspired by charlatanism, those with no scientific basis and which promise patients a cure, and those illusory or insufficiently proven procedures proposed as effective” (Art. 26.2). In addition to the obligation for information to be rigorous, it must be transmitted through the appropriate channels (Art 37.3). Public controversy shall be avoided; disagreements shall be resolved at the professional or associate level (Art. 38). The physician shall not participate in any activity that involves manipulation or hiding of information (Art. 59.4). Art. 64.3 establishes actions as contrary to deontology: “(a) making known in a premature or sensationalist way procedures of efficacy that have not yet been demonstrated or exaggerating this […] (g) Advertising or promoting a product without sufficient scientific support or with insufficient information about it”. Additionally, Art. 65.3: “medical advertising must be objective, prudent, and truthful, so that it does not raise false hopes or spread unfounded concepts”.

In summary, as has been repeatedly stated in case law [46], the physician is not responsible for the outcome, but for performing his or her professional duties in accordance with good practice and *lex artis ad hoc* and this includes the dissemination of only true scientific information through the appropriate channels, and never sowing confusion, covering up, or disguising criticism of other professionals or their work and results.

Consequently, there are legal procedures for the prosecution of professionals who spread FN that can potentially affect health. In addition to the procedures established by the Medical Association, there may also be civil and even criminal proceedings against misinformation provided by healthcare professionals. If it is a result of a contractual relationship between the physician and the client, the Civil Code (Art. 1101) may be of application: “those who, in the fulfilment of their obligations, are guilty of fraudulent, negligent, or delayed action, and those who in any way contravene these obligations, shall be liable for compensation”. If the FN is broadcast free of charge by the health professional and a contractual relationship between him/her and the recipient cannot be invoked, there may still be a proceeding based on Article 1902 of the Civil Code: “the one who, by action or omission, causes damage to another, intervening guilt or negligence, is obliged to repair the damage caused”.

However, it is understandable that given the speed of the development of COVID-19 news, the flourishing of FN may have permeated even to professionals, but it is a must that information publicized by them be evidence based and supported by scientific evidence. These professionals have obligations under the code of ethics and run the risk of being subject to legal actions for non-compliance with the good practices of *lex artis ad hoc.*

Regarding the option of compulsory vaccination, we must look at how this could be achieved. It could be based on the following rationale.

In Spain, Organic Law 3/1986, of 14 April, on Special Measures in the Field of Public Health [47] Article 2, states: “the corresponding health authorities may take measures for examination, treatment, hospitalization, or control when there is reasonable evidence to suggest that the health of the population is endangered by the particular health status of a person or group of persons or by the health conditions in which an activity is carried out” and also Law 41/2002, of 14 November, specifies the basic regulation of patient autonomy and of rights and obligations regarding clinical information and documentation [44] in Article 9.2: “ (a) when there is a risk to public health due to health reasons established by law.” The legal support may be increased by a specific amendment concerning the possibility of introducing a vaccine or compulsory treatments when there may be beneficial effects for all citizens, such as in a pandemic or similar.

Compulsory vaccine regulation would pass the proportionality test as required by the international agreements signed by states in the European Convention on Human Rights [33], since Article 8.2 states: “There shall be no interference by a public authority with the exercise of this right except such as is in accordance with the law and is necessary in a democratic society in the interests of national security or public safety […]” In the case of opposition for religious reasons, in concordance with Art 9.2: “Freedom to manifest one’s religion or beliefs shall be subject only to such limitations as are prescribed by law and are necessary in a democratic society in the interests of public safety, for the protection of public order, health, or morals, or for the protection of the rights and freedoms of others”. For emergency situations, Article 15 could be also applicable: “Derogation in time of emergency: in time of war or other public emergency threatening the life of the nation, any high contracting party may take measures derogating from its obligations under this convention to the extent strictly required by the exigencies of the situation, provided that such measures are not inconsistent with its other obligations under international law”.

In the Charter of Fundamental Rights of the European Union (2000/C 364/01) [48], compatibility may be found in Article 7, in Article 24 when the risk could affect children, and in Article 35 related to health protection.

However, should the vaccine be mandatory? In in a country with an advanced democratic system, the freedom and independence of the citizen to decide (agency, autonomy) is fundamental. Compulsory vaccination would create several grey areas and issues, starting with what a compulsory vaccine is, and issues such as what to do in the case of a person refusing mandatory vaccination, but offering instead to become isolated at home without external contacts. Moreover, a compulsory vaccination mandate would require, to be legally sustained, backing by extensive studies, including all age groups and diffusion of the scientific information prior to becoming part of the medical *praxis and lex artis ad hoc*, in addition to passing the proportionality test, as has been done with lockdown parity argument. Compulsory vaccination, according to good governance, requires the support of citizens, something that is hard to obtain at this point [4,5,6,9,10,11].

It seems that currently the only reasonable approach to COVID-19 vaccination is to promote it as a civic duty, rather than to establish mandatory treatment and penalties for not vaccinating. Adherence could be implemented with vaccine requirements in certain cases, such as for certain jobs in the public sector with frequent contact with the public (e.g., public employees, healthcare workers, police, food handlers, etc.), or as part of the requirements for admission to other work or travel.

In addition to the individual health benefits described, there are other potential externalities associated with a successful vaccination program, including benefits to the economy (avoiding productivity losses), and in avoiding the cost of delayed assistance for other diseases, the publicizing of which may help to increase adherence to vaccination [49].

## 5. Conclusions

Firstly, to obtain gains in health and costs, it is necessary to ensure that the COVID-19 vaccine is safe, without significant side effects, and with a durable immunity effect; however, confidence in the vaccine seems to be jeopardized by disinformation. Scientific data, rigorously analysed by prestigious professionals, must be disseminated to counterbalance the numerous hoaxes circulating in the media and social networks. Full information (together with the active collaboration of citizens) is not only a right but remains the first and cornerstone active measure in promoting a safe vaccination process to provide immunity to the community, including providing the latest scientific results to health professionals as a key element in the diffusion of true data. The relatively low perceived risk for COVID-19 is also another element in the reluctance to be vaccinated [23,50,51,52]. There are also additional approaches for psychological management and resilience, sometimes by novel means such as spot advertising [53], which open up possibilities for behavioural science approaches to the analysis of motivations and their management [54,55,56].

Secondly, for FN itself, following the information gathered from surveys [14], the main role in stopping it, according to the good governance approach, is not for governmental authorities, although new advances in legislation favour collaboration with the media. There is even a small percentage of respondents who consider FN as a right. However, FN can lead to life-threatening behaviours and, therefore, joint action is needed to keep freedom of information, while, at the same time, preserving health. The development of new tools is urgently needed. These could include deep learning techniques and natural language processing, developing systems easily integrated into internet browsers and social networks and providing immediate evidence of whether that statement, news, or content is true or possible misinformation and generating a sort of identifier (possibly in a similar way as the “likes”) on the veracity of the information and its risk for health, or any other method to maintain the right of expression without endangering public health. In any case, the problem is complex and will require finding solutions that both respect freedom of information and guarantee health. A systematic control of information only by the government runs the risk of political bias and it is not the option preferred by citizens, as expressed in surveys.

Thirdly, the case of FN spread by registered professionals (RNs, MDs, etc.) is particularly critical, as, according to the surveys, the news publicized by professionals is considered as highly trustworthy. Users are increasingly looking for health information in the media, and the media is seen as a way of providing information to be promoted by medical associations [3]. New regulations, probably through professional associations, may be required to enhance compliance with the code of ethics, in case of news going against medical praxis, published by associated health professionals.

However, again, prior to promoting punitive actions and the active circulation and updating of scientific data, training and evidence-based continuous education among the professionals promoted by the Medical Association, universities, and other academic institutions seem a reasonable first step.

In conclusion, although, in extreme circumstances, there may be legal possibilities for the compulsory administration of vaccines and for fighting against misinformation when it could undermine public health, particularly when promoted by healthcare professionals, rather than direct and exclusive administrative action, the options preferred by citizens, as expressed in surveys, seem to be for proactive and educative actions of promotion of COVID-19 vaccination as the citizens’ duty, and fighting against FN through information and collaborative plans at governmental level, associations, and media, taking advantage of a the new legal framework [38].

## References

[1] Newman N., Fletcher R., Schulz A., Andı S., Nielsen R.K. Reuters Institute Digital News Report 2020. https://reutersinstitute.politics.ox.ac.uk/sites/default/files/2020-06/DNR_2020_FINAL.pdf.

[2] Harvard Busienss School (2020). Merck CEO Ken Frazier Discusses a COVID Cure, Racism, and Why Leaders Need to Walk the Talk. Blog Post,.

[3] De las Heras-Pedrosa C., Rando-Cueto D., Jambrino-Maldonado C., Paniagua-Rojano F.J. (2020). Exploring the social media on the communication professionals in public health. Spanish official medical colleges case study. Int. J. Environ. Res. Public Health.

[4] Parkhurst J. (2017). The Politics of Evidence: From Evidence-Based Policy to the Good Governance of Evidence.

[5] Page B.I., Shapiro R.Y. (1983). Effects of Public Opinion on Policy. Am. Polit. Sci. Rev..

[6] Matti S. (2009). Exploring Public Policy Legitimacy: A Study of Belief-System Correspondence in Swedish Environmental Policy.

[7] Stelmach J., Brozek B. (2006). Methods of Legal Reasoning.

[8] Feibleman J.K. (1985). Justice, Law and Culture.

[9] Sigma, OECD-EU Public Opinion Surveys as Input to Administrative Reform. http://www.oecd.org/officialdocuments/publicdisplaydocumentpdf/?cote=CCNM/SIGMA/PUMA(98)48&docLanguage=En.

[10] Soroka S.N., Wlezien C., Courtney J.C., Smith D.E. (2010). Public Opinion and Public Policy. The Oxford Handbook of Canadian Politics.

[11] Jacobs L.R. (1992). The recoil effect: Public opinion and policymaking in the U.S. and Britain. Comp. Polit..

[12] Fleiss J.L., Levin B., Paik M.C. (2003). Statistical Methods for Rates and Proportions.

[13] Statista The Statistics Portal for Market Data, Market Research and Market Studies. https://www.statista.com/.

[14] European Commission Flash Eurobarometer 464: Fake News and Disinformation Online. https://op.europa.eu/en/publication-detail/-/publication/2d79b85a-4cea-11e8-be1d-01aa75ed71a1/language-en.

[15] Larson H., de Figueiredo A., Karafillakis E., Rawal M. State of Vaccine Confidence in the EU 2018. https://ec.europa.eu/health/sites/health/files/vaccination/docs/2018_vaccine_confidence_en.pdf.

[16] IPSOS Global Attitudes on a COVID-19 Vaccine, 2020. https://www.ipsos.com/sites/default/files/ct/news/documents/2020-09/global-attitudes-on-a-covid-19-vaccine-ipsos-survey-for-wef-2020.pdf.

[17] Center of Sociological Researches Effects and Consequences of the Coronavirus (II): Advance Results. Tabulation by Sociodemographic Variables. http://datos.cis.es/pdf/Es3302marMT_A.pdf.

[18] Neumann-Böhme S., Varghese N.E., Sabat I., Barros P.P., Brouwer W., van Exel J., Schreyögg J., Stargardt T. (2020). Once we have it, will we use it? A European survey on willingness to be vaccinated against COVID-19. Eur. J. Heal. Econ..

[19] Gallup-Knight Foundation: Americans see Media As Key to Democracy But Increasingly Don’t Trust It. https://knightfoundation.org/press/releases/gallup-knight-foundation-report-americans-see-media-as-key-to-democracy-but-increasingly-don-t-trust-it/.

[20] Monmouth University (2018). “Fake News” Threat to Media; Editorial Decisions, Outside Actors at Fault. Blog Post,.

[21] (2020). Insider NJ Monmouth National—America Takes Sides in Social Media War. Press Release,.

[22] BBVA Foundation International Study of Values. Part One: Values and Attitudes in Europe about the Public Sphere.

[23] Communications Guarantee Authority (AGCOM) Coronavirus Special Reports. https://www.agcom.it/osservatorio-sulla-disinformazione-online.

[24] YouGov|Italia Which Media Are Responsible for Spreading False or Inaccurate Information Regarding the Coronavirus (COVID-19) and Its Impact?. https://www.statista.com/statistics/1111877/opinions-on-fake-news-sources-about-the-coronavirus-by-macro-region-italy/.

[25] Home-Ofcom Covid-19 News and Information: Consumption and Attitudes. https://www.ofcom.org.uk/research-and-data/tv-radio-and-on-demand/news-media/coronavirus-news-consumption-attitudes-behaviour.

[26] World Health Organisation How to Report Misinformation Online. https://www.who.int/campaigns/connecting-the-world-to-combat-coronavirus/how-to-report-misinformation-online?gclid=EAIaIQobChMIj_bv-tqx7QIV5hoGAB1REwetEAAYASAAEgLpjPD_BwE.

[27] European Centre for Disease Prevention and Control Covid-19 Pandemic. https://www.ecdc.europa.eu/en/covid-19-pandemic.

[28] European Commission Fighting Disinformation. https://ec.europa.eu/info/live-work-travel-eu/coronavirus-response/fighting-disinformation_en.

[29] European Commission Tackling Online Disinformation. https://ec.europa.eu/digital-single-market/en/tackling-online-disinformation.

[30] European Commission Funded Projects in the Fight against Disinformation. https://ec.europa.eu/info/live-work-travel-eu/coronavirus-response/fighting-disinformation/funded-projects-fight-against-disinformation_en.

[31] European Commission Social Observatory for Disinformation and Social Media Analysis. https://cordis.europa.eu/project/id/825469.

[32] Head of State (1978). Spanish Constitution. BOE.

[33] Council of Europe European Court of Human Rights European Convention on Human Rights; 1950 with Ammends. https://www.echr.coe.int/documents/convention_eng.pdf.

[34] Renda A. The Legal Framework to Address “Fake News”: Possible Policy Actions at the EU Level. https://www.europarl.europa.eu/RegData/etudes/IDAN/2018/619013/IPOL_IDA(2018)619013_EN.pdf.

[35] Brovdiy Y. (2020). Disinformation in Times of COVID-19: Reinforcing the Responses of the European Union and the United States. CEPOB Coll. Eur. Policy Br..

[36] European Commission Coronavirus: EU Strengthens Action to Tackle Disinformation. https://ec.europa.eu/commission/presscorner/detail/en/ip_20_1006.

[37] EUvsDisinfo (2020). EEAS Special Report Update: Short Assessment of Narratives and Disinformation around the Covid-19 Pandemic (Update May–November). Blog Post,.

[38] Procedimiento de actuación contra la desinformación aprobado por el Consejo de Seguridad Nacional (2020). [Procedure for Action Against Disinformation, National Security Council. National Security Council] Order PCM/1030/2020. BOE.

[39] European Council Fighting Disinformation—COVID-19 Vaccines. https://www.consilium.europa.eu/en/policies/coronavirus/fighting-disinformation/.

[40] United Nations General Assembly (1947). Calling of an International Conference on Freedom of Information. https://digitallibrary.un.org/record/209774?ln=es.

[41] UN General Assembly (1948). Universal Declaration of Human Rights. https://www.un.org/en/ga/search/view_doc.asp?symbol=A/RES/217(III).

[42] World Medical Association (1948). WMA Declaration of Geneva. https://www.wma.net/policies-post/wma-declaration-of-geneva/.

[43] Hendriks A. (1997). Convention for the protection of human rights and dignity of the human being with regard to the application of biology and medicine: Convention on human rights and biomedicine. Eur. J. Health Law.

[44] Head of State (2002). Law 41/2002, basic regulation of patient autonomy, and of rights and obligations regarding clinical information and documentation. BOE.

[45] Council of Official Medical Associations Code of Medical Ethics Guide to Medical Ethics. https://www.cgcom.es/sites/default/files/codigo_deontologia_medica.pdf.

[46] Spanish Supreme Court Case-law STS 1639/2016—ECLI: ES:TS:2016:1639. http://www.poderjudicial.es/search/AN/openCDocument/f9caf3b37c843044ddaedeee43551672cfe231e21f03965a.

[47] Head of State (1986). Organic Law 3/1986, of 14 April, on Special Measures in the Field of Public Health. BOE.

[48] European Union (2000). Charter of Fundamental Rights of the European Union. https://eur-lex.europa.eu/eli/treaty/char_2012/oj.

[49] Roope L.S.J., Buckell J., Becker F., Candio P., Violato M., Sindelar J.L., Barnett A., Duch R., Clarke P.M. (2020). How Should a Safe and Effective COVID-19 Vaccine be Allocated? Health Economists Need to be Ready to Take the Baton. Pharm. Econ. Open.

[50] Ding Y., Du X., Li Q., Zhang M., Zhang Q., Tan X., Liu Q. (2020). Risk perception of coronavirus disease 2019 (COVID-19) and its related factors among college students in China during quarantine. PLoS ONE.

[51] Dryhurst S., Schneider C.R., Kerr J., Freeman A.L.J., Recchia G., van der Bles A.M., Spiegelhalter D., van der Linden S. (2020). Risk perceptions of COVID-19 around the world. J. Risk Res..

[52] Wise T., Zbozinek T.D., Michelini G., Hagan C.C., Mobbs D. (2020). Changes in risk perception and self-reported protective behaviour during the first week of the COVID-19 pandemic in the United States. R. Soc. Open Sci..

[53] Olivares-Delgado F., Iglesias-Sánchez P.P., Benlloch-Osuna M.T., de las Heras-Pedrosa C., Jambrino-Maldonado C. (2020). Resilience and Anti-Stress during COVID-19 Isolation in Spain: An Analysis through Audiovisual Spots. Int. J. Environ. Res. Public Health.

[54] Bavel J.J.V., Baicker K., Boggio P.S., Capraro V., Cichocka A., Cikara M., Crockett M.J., Crum A.J., Douglas K.M., Druckman J.N. (2020). Using social and behavioural science to support COVID-19 pandemic response. Nat. Hum. Behav..

[55] Baldassarre A., Giorgi G., Alessio F., Lulli L.G., Arcangeli G., Mucci N. (2020). Stigma and discrimination (Sad) at the time of the SARS-CoV-2 pandemic. Int. J. Environ. Res. Public Health.

[56] Betsch C. (2020). How behavioural science data helps mitigate the COVID-19 crisis. Nat. Hum. Behav..

